# Effect of chronic unpredictable stress on mice with developmental under-expression of the Ahi1 gene: behavioral manifestations and neurobiological correlates

**DOI:** 10.1038/s41398-018-0171-1

**Published:** 2018-07-02

**Authors:** Gilly Wolf, Tzuri Lifschytz, Hagar Ben-Ari, Pavel Tatarskyy, Tirzah Kreisel Merzel, Amit Lotan, Bernard Lerer

**Affiliations:** 1 Biological Psychiatry Laboratory, Hadassah-Hebrew University Medical Center, Jerusalem, Israel; 2 Hadassah BrainLabs - National Knowledge Center for Research on Brain Diseases, Hadassah-Hebrew University Medical Center, Jerusalem, Israel; 3 Departments of Psychology and Life Sciences, School of Sciences, Achva Academic College, Be’er Tuvia, Israel; 4 Department of Developmental Biology and Cancer Research, Hadassah- Hebrew University Medical School, Jerusalem, Israel

## Abstract

The Abelson helper integration site 1 (Ahi1) gene plays a pivotal role in brain development and is associated with genetic susceptibility to schizophrenia, and other neuropsychiatric disorders. Translational research in genetically modified mice may reveal the neurobiological mechanisms of such associations. Previous studies of mice heterozygous for Ahi1 knockout (Ahi1+/−) revealed an attenuated anxiety response on various relevant paradigms, in the context of a normal glucocorticoid response to caffeine and pentylenetetrazole. Resting-state fMRI showed decreased amygdalar connectivity with various limbic brain regions and altered network topology. However, it was not clear from previous studies whether stress-hyporesponsiveness reflected resilience or, conversely, a cognitive-emotional deficit. The present studies were designed to investigate the response of Ahi1+/− mice to chronic unpredictable stress (CUS) applied over 9 weeks. Wild type (Ahi1+/+) mice were significantly affected by CUS, manifesting decreased sucrose preference (*p* < 0.05); reduced anxiety on the elevated plus maze and light dark box and decreased thigmotaxis in the open field (*p* < 0.01 0.05); decreased hyperthermic response to acute stress (*p* < 0.05); attenuated contextual fear conditioning (*p* < 0.01) and increased neurogenesis (*p* < 0.05). In contrast, Ahi1+/− mice were indifferent to the effects of CUS assessed with the same parameters. Our findings suggest that Ahi1 under-expression during neurodevelopment, as manifested by Ahi1+/− mice, renders these mice stress hyporesponsive. Ahi1 deficiency during development may attenuate the perception and/or integration of environmental stressors as a result of impaired corticolimbic connectivity or aberrant functional wiring. These neural mechanisms may provide initial clues as to the role Ahi1 in schizophrenia and other neuropsychiatric disorders.

## Introduction

Abelson helper integration site-1 (Ahi1) is a neurodevelopmental gene that encodes a cytoplasmic adaptor protein containing a coiled-coil domain, seven WD40 repeats and an SH3 domain^1^. Ahi1 is located on chromosome 6q23 in humans and chromosome 10 in mice. Expressed in the embryonic hindbrain and forebrain, Ahi1 is crucial for cerebellar and cortical development^2^. The combination of protein-binding domain motifs indicates that Ahi1 is involved in protein–protein interactions^1,3^. Ahi1 regulates cilium formation via its interaction with Rab8a, a small GTPase critical for polarized membrane trafficking^4^.

Mutations in Ahi1 that lead to loss of function underlie Joubert syndrome, a rare, severe autosomal disorder characterized by multi-system abnormalities including low muscle tone, abnormal eye movements, severe motor deficits, and cognitive impairment^3,5^. Our group demonstrated association of the locus with susceptibility to schizophrenia^6,7^. Study of a homogenous sample of Arab, Israeli families identified several single nucleotide polymorphisms in the 6q23.3 region that were associated with the disorder^7^. These associations were replicated in Icelandic^8^ and large European samples^9^ and were supported by findings in cohorts of German and Spanish^10^ and North Indian patients^11^ and by QTL analysis in independent European samples^12^. Studying Ahi1 expression levels in lymphoblasts from schizophrenia patients, we found reduced expression in patients with early onset of the disorder^13^, leading us to suggest that reduced Ahi1 levels may be associated with susceptibility to schizophrenia. Association of Ahi1 with autism^14,15^ and bipolar disorder^16^ has also been reported.

We have used mice heterozygous for a constitutional knockout of the Ahi1 gene (Ahi1+/− mice) to conduct translational studies of the Ahi1 gene and to further elucidate the potential role of the gene in psychiatric disorders. Homozygous knockout mice (Ahi1−/−) manifest severe neurological deficits, that serve as a suitable model for Joubert syndrome^17^, but not for schizophrenia or other psychiatric phenotypes. Consistent with the neurodevelopmental role of Ahi1, we showed that newborn Ahi1+/− mice manifest a 65% reduction in brain Ahi1 levels compared to Ahi1+/+mice^18^. Ahi1+/− mice do not manifest sensory or motor impairment and develop normally. Notwithstanding our finding of association with schizophrenia, we did not observe differences between Ahi1+/+ and Ahi1+/− mice on paradigms that focus on what are considered as rodent-equivalent of positive symptoms such as prepulse inhibition (PPI) or MK-801-induced hyperlocomotion^18^. However, extensive behavioral phenotyping of Ahi1+/− mice demonstrated decreased anxiety and glucocorticoid response to stressful stimuli on several behavioral tests. These findings were observed in the context of a normal HPA-axis function, as demonstrated by an expected corticosterone response to centrally-acting anxiogenic compounds, such as caffeine and pentylenetetrazole, that could bypass potential defects in recognition of environmental stress^18^. Further investigation employing resting-state fMRI indicated decreased amygdalar connectivity with various limbic brain areas including the ventral hippocampus, lateral entorhinal cortex, and ventral tegmental area.

The behavioral phenotype of Ahi1+/− mice and their altered brain connectivity suggest that Ahi1 deficiency during neurodevelopment could attenuate the effect of environmental cues that normally evoke stress and anxiety. We suggested that altered neuronal connectivity of Ahi1 deficient mice that results in impaired integrative processing of environmental cues, leading to blunted threat detection, may underlie their stress hyporesponsiveness^18,19^. Thus, the low anxiety profile of Ahi1+/− mice could ostensibly reflect stress resilience while it actually derives from a cognitive-emotional deficit that may have relevance to the pathogenesis of neuropsychiatric disorders in which the gene has been implicated^6–10,12,14–16^. However, our previous studies^18,19^, which showed hyporesponsiveness of Ahi1+/− mice when exposed to acute stress did not directly address this important conceptual difference.

In an attempt to better position their ostensible stress hyporesponsiveness in its relevant (patho)physiological context (i.e., resilience as opposed to cognitive-emotional deficit), we exposed Ahi1+/− mice to a protocol of chronic unpredictable stress (CUS) that consisted of various psychological stressors administered in random order over a period of 9 weeks. CUS paradigms are based on the chronic, mild stress protocol of Willner et al.^20^ that has been shown to induce anhedonia (e.g.,^21,22^) along with decreased hippocampal neurogenesis^23,24^. With respect to anxiety, data are less consistent. Increased anxiety was originally reported by Wilner^20^ and also observed by others^25,26^. However, several groups^27–29^ including ours^30^ have noted a decrease in anxiety-related behavior in rodents after exposure to CUS, while others reported no significant change^31,32^. We hypothesized that CUS would induce anhedonia in Ahi1+/+ mice with alterations in neurogenesis and would not affect anxiety, whereas Ahi1+/− mice that were previously characterized as hyporesponsive on exposure to acute stress, would be less affected or even indifferent to the CUS protocol.

## Materials and methods

### Establishment of Ahi1+/− mouse line reproduction colony

The generation of Ahi1+/− mice and their Ahi1+/+ littermates was described previously^18^. Briefly, the founders of the colony were chimeric mice carrying a gene trap vector preventing the expression of the Ahi1 gene. These mice were crossed with WT mice (C57BL/6) to produce heterozygous Ahi1 knockout mice (Ahi1+/−). Ahi1+/− mice were again back crossed to WT (Ahi1+/+) females for over 20 generations before using animals in the current experiments. In generating animals for experiments, Ahi1+/− males and Ahi1+/+ females were bred to obtain Ahi1+/− and Ahi1+/+ animals as littermates. All experiments comply with the ARRIVE guidelines, were approved by the Ethics Committee of The Hebrew University Authority for Biological and Biomedical Models and were carried out in accordance with the National Institutes of Health Guide for the Care and Use of Laboratory animals (NIH Publications No. 8023, revised 1978).

### Animals

Seventy-three male mice aged 9 weeks at the beginning of the study were employed. 39 Ahi1+/+ mice were employed, 24 of whom randomly assigned as control and 15 randomly assigned to chronic unpredictable stress protocol (CUS, see below). 34 Ahi1+/− mice were employed, 17 randomly assigned as control and 17 randomly assigned to CUS protocol. The number of mice used in the study was the maximum that was approved by the Hebrew University Committee on Animal Use and Care, and was high enough to ensure statistical power of the analyses that were used. According to ethical regulations, 8 mice (1 Ahi1+/+ control, 2 Ahi1+/+ CUS, 3 Ahi1+/− control and 2 Ahi1+/− CUS) that were severely wounded due to intracage violence were excluded from the study and immediately terminated.

### Chronic unpredictable stress (CUS) protocol

Mice were exposed to a protocol of various environmental stressors in random order. Stressors included illumination during the dark phase of the day (6–9 h), cage tilt (45°, 4–6 h), overnight soiled cage bedding (14 h), 1–5 min restraint (in 50 ml falcon tube), white noise (80 db, 2–6 h), and flashing lights (4–8 h). After 5 full weeks of exposure to the stress protocol, mice underwent a 3-week battery of behavioral, affective and cognitive phenotyping with the stress protocol ongoing. Supplementary Fig. 1 shows the CUS protocol and timeline of the experiment.

### Behavioral tests

#### Procedure

Mice of the four different genotype and CUS exposure groups (Ahi1+/+ control, Ahi1+/+ CUS, Ahi1+/− control, Ahi1+/− CUS) were grouped 2–3 mice per cage and baseline measures of the social exploration (SE) and sucrose preference test (SPT) were performed. Then, CUS mice underwent the CUS protocol while control mice remained in their regular setting. All the mice were weighed weekly and were similarly handled during cage maintenance. After 5 weeks all mice underwent a 3-week assessment battery consisting of tests evaluating anxious, depressive, and cognitive phenotypes. Rotarod, SE, sucrose preference, and stress-induced hyperthermia tests were performed manually by experimenters blind to the group allocation of the animals in a dedicated SPF-standard room. All other behavioral experiments were recorded and analyzed using the Ethovision 10 system. The order and timing of the tests is provided in the Supplementary Information.

#### Sucrose preference test (SPT)

The SPT was conducted twice: first, at baseline prior to any manipulation, and second, as part of the behavioral-cognitive battery of tests used to assess the effects of stress. To avoid isolation stress sucrose preference was measured per cage, reflecting the average consumption of its residents (2–3 mice of the same genotype and treatment).The first SPT required introduction of the sucrose solution prior to the actual testing; hence it included 2 days of habituation during which mice were given free access to water and 2% sucrose solution followed by 2 test days during which the amounts of water and sucrose solution consumed per cage were measured every 24 h. In the second SPT mice were given free access to water and 2% sucrose solution for 24 h and the amounts of water and sucrose solution consumed per cage were measured. Sucrose preference was calculated as the ratio of sucrose consumed divided by the total liquid consumption.

#### Open field test (OF)

Mice were placed in the corner of a 50*50*33 cm (height) arena, and were allowed to freely explore the arena for 6 min. The center of the arena was defined as a 25*25 cm square in the middle of the arena. Velocity and time spent in the center and arena circumference were measured.

#### Elevated plus maze (EPM)

The test apparatus consists of two open arms (30*5 cm) bordered by a 1 cm high rim across from each other and perpendicular to two closed arms bordered by a rim of 16 cm, all elevated 75 cm from the floor. Mice were entered into the maze and were allowed to explore it for 5 min. Duration of visits in both the open and closed arms were recorded.

#### Light dark box (LDB)

This test is conducted using an apparatus consisting of an arena (45*21*21 cm) partitioned into a lighted open compartment (30*21*21 cm) and a dark closed (15*21*21 cm). A small entrance within the compartment partition (5*5 cm) allows each animal to move freely between the chambers. Mice were placed in the light compartment and the duration of their stay in both compartments were recorded.

#### Fear conditioning (FC)

This test is conducted in an apparatus (PanLab) built of a double box (external part and an internal one to which an animal is inserted). The device consists of grid floor capable of transmitting low electrical current over small time intervals, surrounded by black or white walls. During the conditioning part of the test mice were individually placed in the conditioning chamber for 2 min of habituation, followed by two sets of 20 s tone and 2 s 0.5 milliampere foot shock. Thirty seconds of rest followed the second tone/shock set, after which mice were returned to their home cage. Contextual and cued conditioning were tested 24 h later. For the contextual test mice were placed individually in the same conditioning chamber for 5 min. The amount of time mice spent freezing was measured. For the cued test a metal board was placed on the grid floor and the wall color was changed. Mice were individually placed in the chamber for 2.5 min of habituation, followed by 2.5 min in which the same tone that was used in the conditioning was sounded. The amount of time mice spent freezing was measured. There was an interval of at least 2 h between the contextual and cued tests. All tests were fully computerized (Packwin).

#### Stress-induced hyperthermia (SIH)

This test is conducted using a single probe thermometer connected to a specialized rectal probe. Upon insertion, basal rectal temperature was measured, the insertion of the probe representing the first stress exposure. To test the effect of acute stress on rectal temperature the probe was inserted again 10 min later, and rectal temperature was measured.

#### Additional behavioral tests

Methodological details of the forced swim (FST), SE, and novel object recognition (NOR) tests are provided in the Supplementary Information. Tests relevant to schizophrenia such as prepulse inhibition and MK-801-induced hyperlocomotion were not performed since these had shown no effect of acute stress in our prior studies^18^^[,19^.

### Immunohistochemistry

Animals were perfused transcardially with cold phosphate-buffered saline (PBS) followed by 4% formaldehyde and the brains were quickly removed and placed in 4% formaldehyde. After 24 h, the brains were placed in 20% sucrose solution in DDW and then frozen at optimal cutting temperature. Brains were dissected to 50 μm frozen floating sections.

Doublecortin (DCX) staining was performed on frozen floating brain sections. The sections were fixed in methanol, washed twice with PBS and incubated overnight in 1% bovine serum and 0.1% triton in 1XPBS with the primary antibody (Anti-Doublecortin −1 1:3000, Millipore-Temecula, CA, USA) at 4 °C. Sections were then incubated with the secondary antibody (Cy5, donkey anti guinea pig, 1:400; Jackson ImmunoResearch) for 2 h at room temperature (RT) and counter-stained with DAPI (Sigma, Israel). DCX images were captured using an Olympus FV-1000 confocal microscope and camera (Tokyo, Japan). The number of DCX marked cells was manually counted at ×20 in a defined area containing the entire granular cell layer (GCL) of the hippocampus. DCX marked cells were counted twice by an observer blind to age and exposure with the same results. Number of visible DCX-stained cells was divided by the GCL volume. Volume of GCL was calculated as following: (GCL area * number of stacks * stack spacing * µm/pixels^2^)/10^9^.

### Corticosterone assay

Corticosterone levels were measured in serum obtained from mice at sacrifice. Corticosterone level was measured using a corticosterone ELISA kit and protocol (R&D systems, Inc., Minneapolis, USA). (Details provided in Supplementary Information).

### Statistical analysis

Data were analyzed using SPSS 20. Two-way analysis of variance (ANOVA) was performed, with repeated measures when appropriate, followed by univariate tests of simple main effects with Bonferroni correction for post hoc comparisons. Data in figures are given as mean ± standard error of the mean (s.e.m.). Analysis of covariance (ANCOVA) was performed when a significant baseline difference was demonstrated. *P* values < 0.05, two tailed, were regarded as statistically significant. The number of mice (*N*) employed in this study was restricted due to ethical considerations by the Hebrew University Committee on Animal Care and Use. A priori power analysis using G*Power 3.1.9.2 indicated that the *N* approved was large enough to detect moderate to large effect size (*η*^2^ > 0.4) using the statistical analyses that were performed (Two-tailed alpha < 0.05; power 0.8). Sensitivity analysis using G*Power 3.1.9.2 indicated that the sample employed detects an effect size of 0.33 and above (Two-tailed alpha < 0.05; power 0.8).

## Results

### Behavioral and cognitive effects

#### Effect of genotype and CUS on sucrose preference

**Fig. 1:**
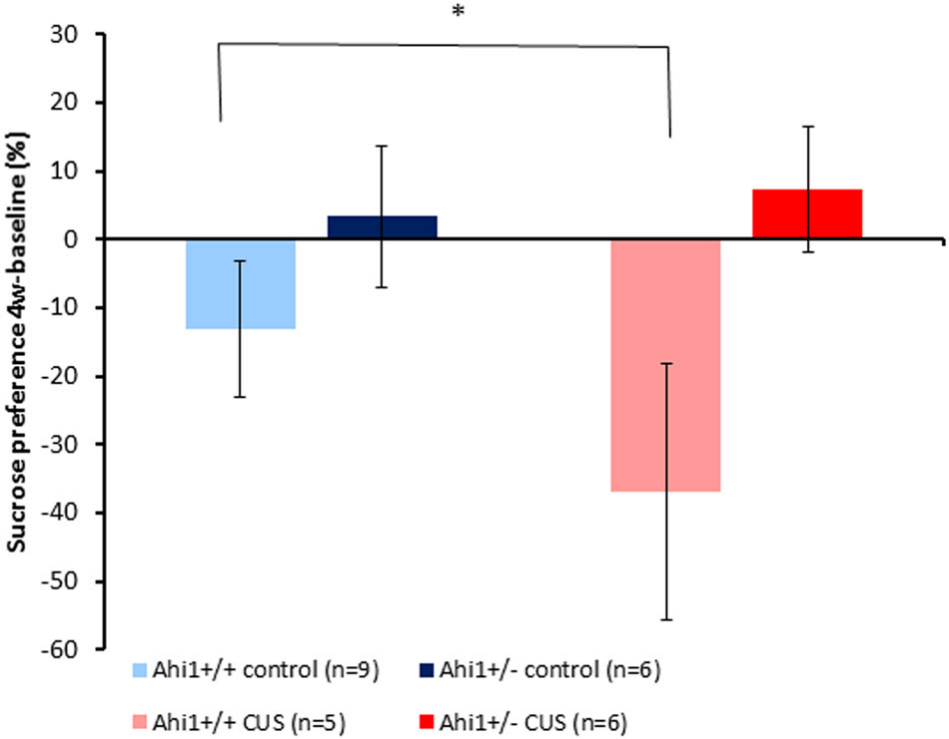
Effect of CUS on sucrose preference. Two-way ANOVA revealed a significant main effect of genotype (*F*[1,22] = 7. 563, *p* = 0.012), indicating a differential effect of CUS on the two genotypes. Post hoc comparisons of simple main effects with Bonferroni correction indicated that CUS-exposed Ahi1+/+ mice displayed decreased sucrose preference compared to Ahi1+/+ controls (*p* < 0.05), but no such difference was demonstrated in CUS-exposed Ahi1+/− mice compared to Ahi1+/− controls. *p < 0.05

Results for the SPT are per cage and reflect the average sucrose consumption of 2–3 mice. There were no differences between groups in sucrose consumption at baseline before exposure to the CUS protocol. CUS exposure resulted in decreased sucrose preference of Ahi1+/+ mice but had no effect on Ahi1+/− mice (Fig. 1). Two-way ANOVA revealed a significant main effect of genotype (*F*[1,22] = 7. 563, *p* = 0.012). Levene’s test of equality of error variances indicated that equality of error variances assumption was sustained (*F*[3,22] = 0.787, *p* = 0.514). Post hoc comparisons of simple main effects with Bonferroni correction indicated that CUS-exposed Ahi1+/+ mice displayed decreased sucrose preference following 5 weeks of CUS compared to Ahi1 +/+controls (*p* < 0.05) at the same time point; no such difference was observed in Ahi1+/− mice compared to Ahi1+/− controls.

#### Effect of genotype and CUS on presence in the center of the arena in the OF

**Fig. 2:**
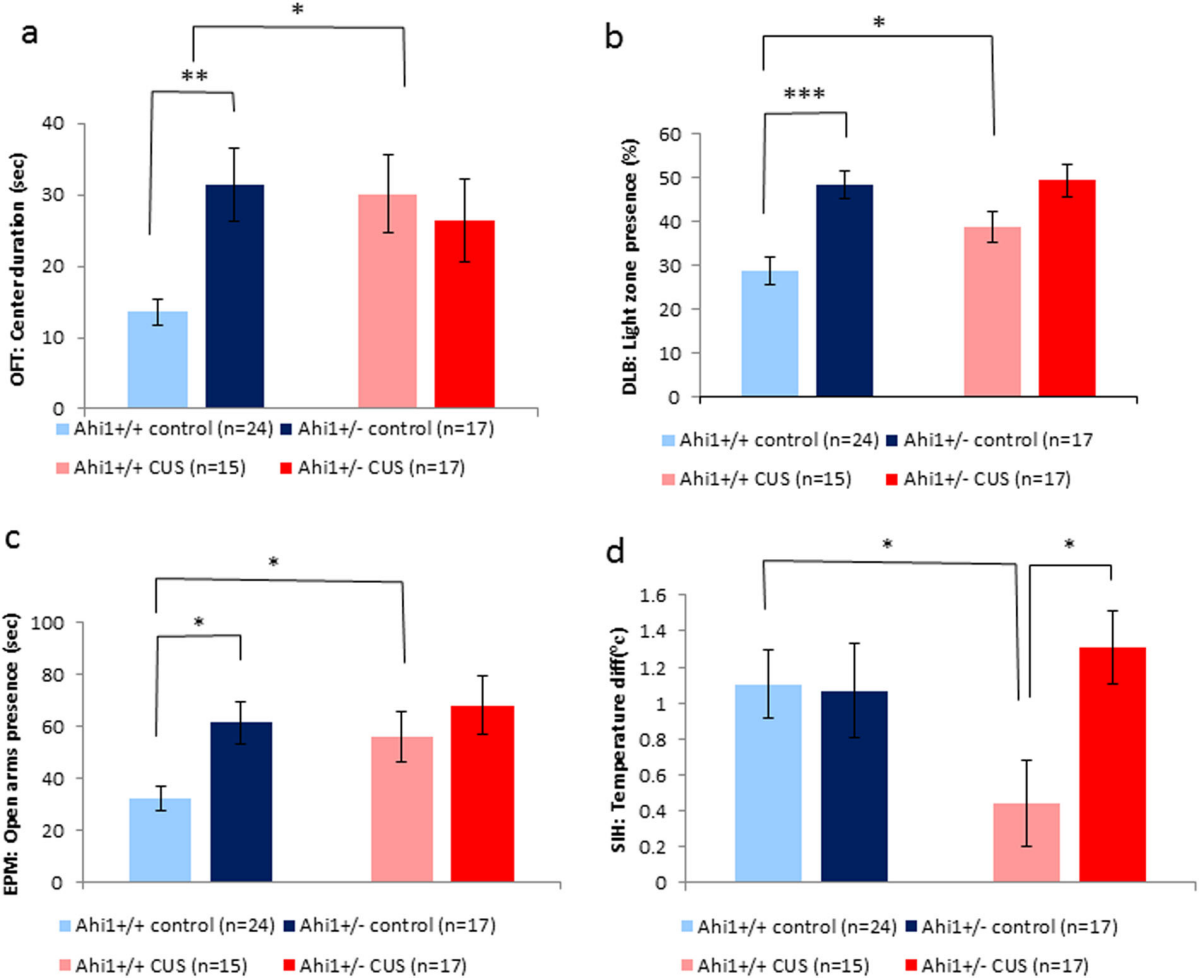
Effect of CUS on anxiety. **a** Effect of CUS on time spent in the center of the open field. Two way ANOVA revealed a significant genotype by CUS interaction (*F*[1,70] = 6.08, *p* = 0.016. Post hoc comparisons of simple main with Bonferroni correction effects indicated a significant difference between Ahi1+/+ and Ahi1+/− control mice (*p* < 0.005), and between CUS-exposed Ahi1+/+ and Ahi1+/+ controls (*p* < 0.01), but not between CUS-exposed Ahi1+/− and Ahi1+/− controls. *p < 0.05, **p < 0.005; **b** Effect of CUS on light-dark box preference. Significant main effect of genotype was detected on two-way ANOVA (*F*[1,70] = 20.18, *p* = 0.00003), indicating that Ahi1+/− mice spent longer in the open area of the dark-light box than Ahi1+/+ mice. Post hoc comparisons of simple main effects with Bonferroni correction revealed that Ahi1+/− control mice spent longer in the light zone than Ahi1+/+ controls (*p* < 0.0001), and CUS-exposed Ahi1+/+ mice spent longer in the light zone than Ahi1+/+ controls (*p* < 0.05), suggesting that the CUS protocol decreased the anxiety of these mice. No such effect was observed in CUS-exposed Ahi1+/− mice. *p < 0.05, ***p < 0.0001; **c** Effects of CUS on time spent in the open arms of the elevated plus maze. Significant main effect of genotype (*F*[1,70] = 6.869, *p* = 0.011) on two-way ANOVA, indicating that Ahi1+/− mice spent longer in the open arms of the elevated plus maze than Ahi1+/+ mice, suggesting reduced anxiety of these mice. Post hoc comparisons of simple main effects with Bonferroni correction indicated that Ahi1+/− mice spent longer in the open arms compared to Ahi1+/+ controls (*p* < 0.01), and that CUS-exposed Ahi1+/+ mice spent longer in the open arms compared to Ahi1+/+ controls (*p* < 0.05). No similar effect was observed in the CUS-exposed Ahi1+/− mice. *p < 0.05 **d** Effect of CUS in the stress-induced hyperthermia test. Two-way ANOVA revealed a significant main effect of genotype (*F*[1,63] = 4.203, *p* = 0.045), indicating that Ahi1+/+ mice manifested attenuated hyperthermia. CUS blunted hyperthermia in Ahi1+/+ mice, but had no effect on Ahi1+/− mice, reflected in a significant CUS by genotype interaction (*F*[1,63] = 5.001, *p* = 0.029). Post hoc tests of simple main effects with Bonferroni correction indicated that CUS-exposed Ahi1+/+ mice displayed a blunted response to acute stress compared to both CUS-exposed Ahi1+/− mice (*p* < 0.05) and Ahi1+/+ controls (*p* < 0.05). There was no significant difference between CUS-exposed Ahi1+/− mice and Ahi1+/− control mice. *p < 0.05

Consistent with our previous observations^18^, Ahi1+/− control mice spent significantly more time in the center of the arena compared to Ahi1+/+ controls. CUS increased the amount of time Ahi1+/+ mice spent in the center of the arena, compared to Ahi1+/+ controls but had no effect on Ahi1+/− mice, reflected in a genotype by CUS interaction (*F*[1,70] = 6.08, *p* = 0.016). Levene’s test of equality of error variances indicated that equality of error variances assumption was not sustained (*F*[3,70] = 5.919, *p* < 0.001). Post hoc comparisons of simple main effects with Bonferroni correction indicated a significant difference between Ahi1+/+ and Ahi1+/− control mice (*p* < 0.005), and between CUS-exposed Ahi1+/+ mice and Ahi1+/+ controls (*p* < 0.01). No significant difference was demonstrated between CUS-exposed Ahi1+/− mice and Ahi1+/− controls. These results suggest that CUS reduces anxiety of Ahi1+/+ mice but has no such effect on Ahi1+/− mice (Fig. 2a). Genotype (*F*[1,70) = 1.731, *p* = 0.193) and CUS exposure (*F*[1,70] = 2.721, *p* = 0.103) had no significant effect on the general motor activity of the mice.

#### Effect of genotype and CUS on presence in the lighted area of dark-light box

Consistent with the result of the OF and our previous findings^18^, Ahi1+/− mice spent longer in the light area of the dark-light box, indicating lower anxiety of these mice. This was reflected in a significant main effect of genotype (*F*[1,70] = 20.184, *p* = 0.00003). Levene’s test of equality of error variances indicated that equality of error variances assumption was sustained (*F*[3,70] = 1.011, *p* = 0.393). Post hoc comparisons of simple main effects with Bonferroni correction revealed that CUS-exposed Ahi1+/+ mice spent longer in the open area of the arena than Ahi1+/+ controls (*p* < 0.05), suggesting that the CUS protocol decreased the anxiety of these mice. No such effect was observed in CUS-exposed Ahi1+/− compared to Ahi1+/− control mice (Fig. 2b).

#### Effects of genotype and CUS on presence in the open arms of the EPM

Consistent with the other anxiety tests performed in this study, Ahi1+/− mice spent longer in the open arms of the EPM, suggesting reduced anxiety of these mice. This was reflected in a significant main effect of genotype (*F*[1,70] = 6.869, *p* = 0.011). Levene’s test of equality of error variances indicated that equality of error variances assumption was sustained (*F*[3,70] = 1.931, *p* = 0.133).Post hoc comparisons of simple main effects with Bonferroni correction indicated that Ahi1+/− control mice spent longer in the open arms compared to Ahi1+/+ controls (*p* < 0.01). CUS-exposed Ahi1+/+ mice spent longer in the open arms compared to Ahi1+/+ controls (*p* < 0.05). No similar elevation was observed in the CUS-exposed Ahi1+/− compared to Ahi1 control mice (Fig. 2c).

#### Effect of genotype and CUS on acute stress response in stress-induced hyperthermia

Ahi1+/− mice manifested increased hyperthermia in the stress-induced hyperthermia test, reflected by a significant main effect of genotype (*F*[1,63] = 4.203, *p* = 0.045) on two-way ANOVA. CUS blunted stress-induced hyperthermia in Ahi1+/+ mice compared to Ahi1+/+ controls, but had no effect on Ahi1+/− mice. This effect was reflected in a significant CUS by genotype interaction (*F*[1,63] = 5.001, *p* = 0.029). Levene’s test of equality of error variances indicated that equality of error variances assumption was sustained (*F*[3,63] = 0.974, *p* = 0.411). Post hoc tests of simple main effects with Bonferroni correction indicated that CUS-exposed Ahi1+/+ mice displayed blunted response to acute stress compared to both Ahi1+/+ controls (*p* < 0.05) and CUS-exposed Ahi1+/− mice (*p* < 0.05). There was no significant difference between CUS-exposed Ahi1+/− and Ahi1+/− control mice (Fig. 2d).

#### Effect of genotype and CUS on contextual and cued FC

**Fig. 3:**
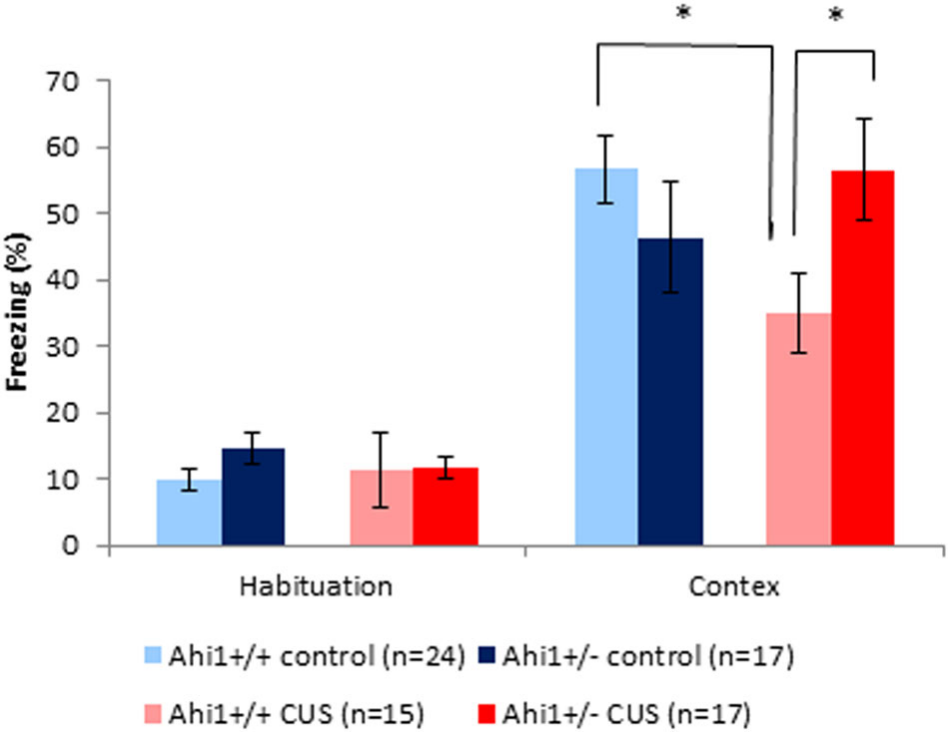
Effect of CUS on fear conditioning. A significant genotype by CUS interaction was observed on two-way ANOVA, demonstrating that CUS-exposed Ahi1+/+ mice displayed an attenuated freezing response to the conditioning chamber following fear training compared to Ahi1+/+ controls and CUS-exposed Ahi1+/− mice (*F*[1,66] = 7.192, *p* = 0.009). Post hoc tests of simple main effects with Bonferroni correction indicated that CUS-exposed Ahi1+/+ mice displayed diminished freezing response to the conditioned context compared to both CUS-exposed Ahi1+/− mice (*p* < 0.05) and Ahi1+/+ controls (*p* < 0.05). There was no significant difference between CUS-exposed Ahi1+/− mice and Ahi1+/− control mice. No significant differences were observed on cued freezing response to the conditioned tone. *p < 0.05

CUS-exposed Ahi1+/+ mice displayed an attenuated freezing response to the conditioning chamber following fear training compared to Ahi1+/+ controls and CUS-exposed Ahi1+/− mice, reflected by a significant genotype by CUS interaction (*F*[1,66] = 7.192, *p* = 0.009). Levene’s test of equality of error variances indicated that equality of error variances assumption was sustained (*F*[3,66] = 1.045, *p* = 0.379). Post hoc tests of simple main effects with Bonferroni correction indicated that although all mice displayed increased freezing behavior compared with habituation phase, this increase was diminished in CUS-exposed Ahi1+/+ mice compared to both Ahi1+/+ controls (*p* < 0.05) and CUS-exposed Ahi1+/− mice (*p* < 0.05). There was no significant difference between CUS-exposed Ahi1+/− and Ahi1+/− control mice (Fig. 3). On testing for cued freezing response to the conditioned tone, Ahi1+/+ and Ahi1+/− mice displayed similar freezing response independent of CUS. No significant effect of genotype, CUS, or genotype by CUS interaction was identified on two-way ANOVA.

### Effects of Ahi1 genotype and CUS on neurogenesis

**Fig. 4:**
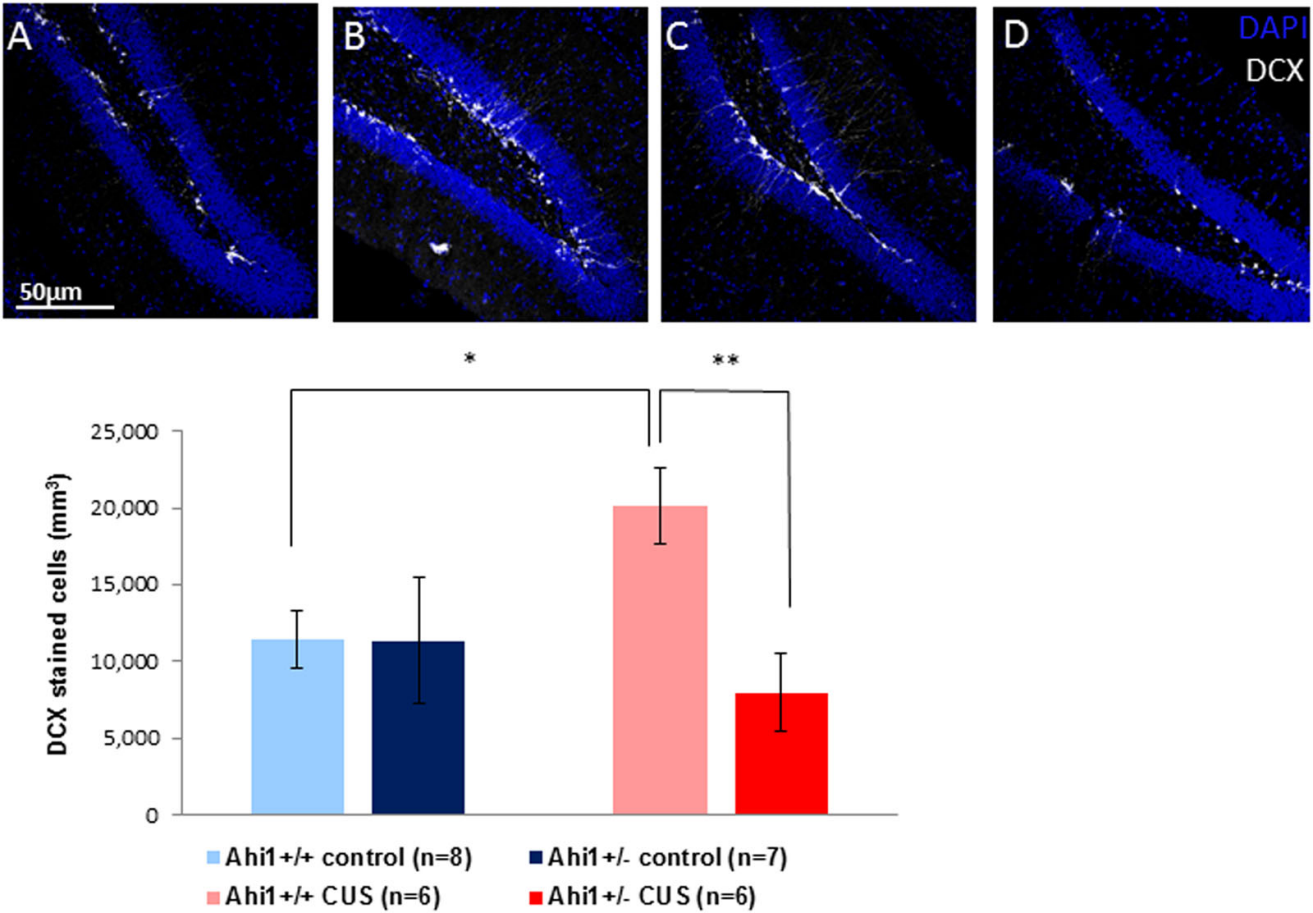
*Top*: Immunofluorescence staining of doublecortin (DCX) in the granule cell layer of the dentate gyrus of hippocampi of Ahi1+/+- Control (**a**, *n* = 8) and Ahi1+/+ CUS mice (**b**, *n* = 6); and, Ahi1+/− Control (**c**, *n* = 7) and Ahi1+/− CUS (**d**, *n* = 6) male mice. Bottom: Bar graph of DCX quantification. Two way ANOVA revealed a significant interaction between genotype and CUS exposure (*F*[1,23] = 5.436, *p* = 0.029). Post hoc tests of simple main effects with Bonferroni correction revealed a significantly higher number of newly formed neurons in CUS exposed Ahi1+/+ mice compared to Ahi1+/+ mice control (*p* = 0.024) and Ahi1+/− mice exposed to CUS (*p* = 0.04). However, no difference in DCX marked cells was seen in Ahi1+/− CUS mice compared to Ahi1+/− control mice

Figure 4 shows the findings obtained by immunofluorescence staining with DCX in the GCL of the hippocampal dentate gyrus. There was a significant effect of genotype (*F* [1,23] = 5.578, *p* = 0.027) and a significant interaction between genotype and CUS exposure (*F*[1,23] = 5.436, *p* = 0.029). Levene’s test of equality of error variances indicated that equality of error variances assumption was sustained (*F*[3,23] = 1.075, *p* = 0.379). Post hoc tests of simple main effects with Bonferroni correction showed a significantly higher number of newly formed neurons in CUS-exposed Ahi1+/+ compared to Ahi1+/+ control mice (*p* = 0.024), and to Ahi1+/− mice exposed to CUS (*p* = 0.04). There was no difference in the number of DCX marked cells in Ahi1+/− mice exposed to CUS and Ahi1+/− mice maintained under control conditions.

### Effects of Ahi1 genotype and CUS on serum corticosterone level

**Fig. 5:**
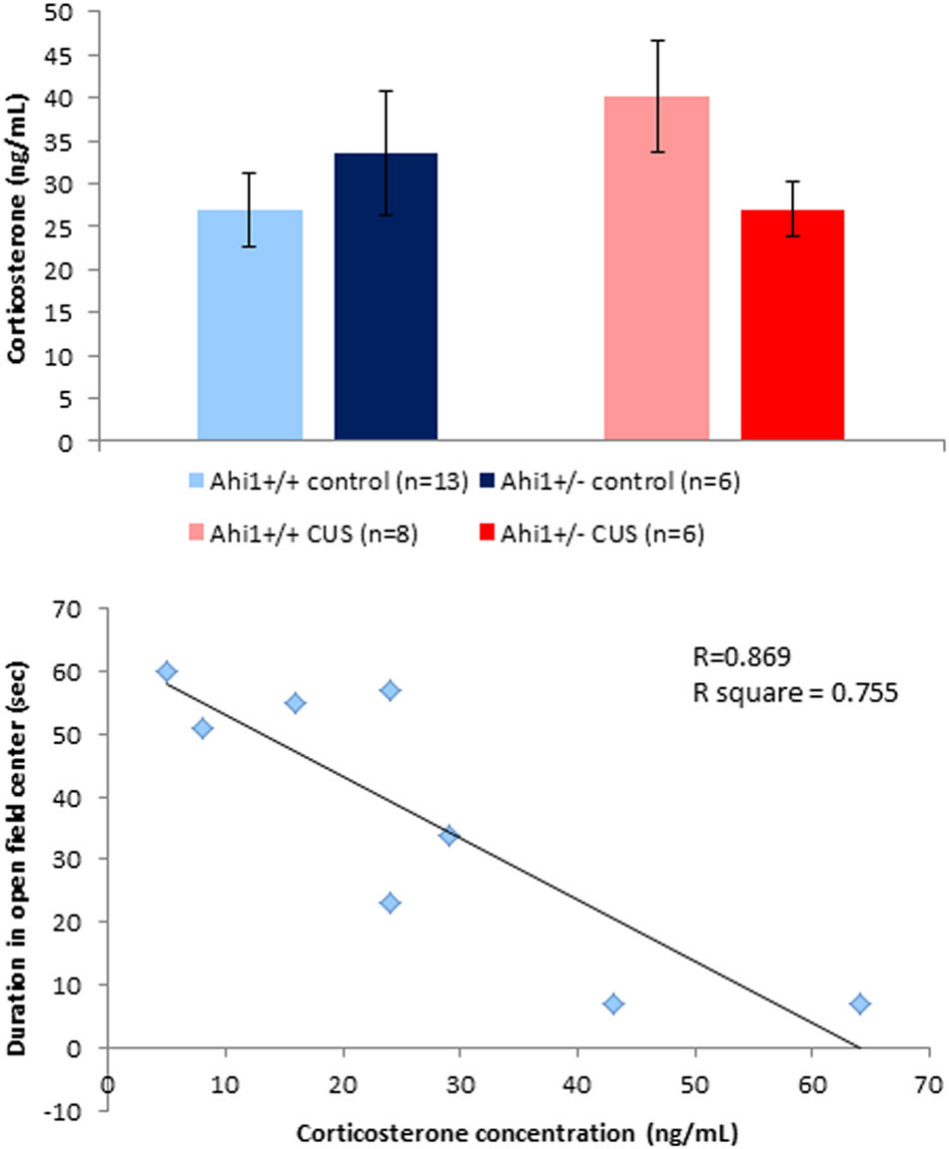
Effect of CUS on corticosterone level. Top: Two-way ANOVA revealed a trend towards genotype by treatment interaction (*F*[1,29] = 3.404, *p* = 0.075). Bottom: Correlation of corticosterone and presence to the center of the open field in CUS-exposed Ahi1+/+ mice. Strong and significant negative correlation (*R*^2^ = 0.755) of corticosterone level and duration in the center of the open field (*F*[1,6] = 18.488, *p* = 0.005), implying that the higher the corticosterone level the shorter is the duration in the center of the arena

There was a trend towards elevation of serum corticosterone in CUS-exposed Ahi1+/+ mice compared to Ahi1+/+ controls, whereas no such effect of CUS was observed in Ahi1+/− mice (genotype by treatment interaction, *F*[1,29] = 3.404, *p* = 0.075; Fig. 5, top). Levene’s test of equality of error variances indicated that equality of error variances assumption was not sustained (*F*[3,33] = 4.273, *p* < 0.05). To determine the relationship between corticosterone level and the behavioral phenotype in the OF, Pearson correlation was computed for values from all mice. There was a strong and significant negative correlation (*R*^2^ = 0.755) of corticosterone level and duration in the center of the open field (*F*[1,6] = 18.488, *p* = 0.005), implying that the higher the corticosterone level the shorter the duration in the center of the arena (Fig. 5, bottom).

## Discussion

In this study we examined the response to CUS of mice heterozygous for knockout of the Ahi1 gene (Ahi1+/− mice) compared to the response of wild type (Ahi1+/+) littermates. We had previously shown that Ahi1+/− mice not subjected to prior stress, displayed significantly lower anxiety levels than wild type Ahi1+/+ mice on a number of anxiety tests as well as functional corticolimbic disconnectivity^18,19^. In the current study we replicated our findings regarding lower levels of anxiety in stress-naïve Ahi1+/− compared to Ahi1+/+ mice in the OF, light-dark box and EPM. We also found a significantly greater duration of SE in Ahi1+/− mice (Supplementary Information).

Our current findings show that exposure to CUS induced decreased sucrose preference, reduced anxiety (manifested on three anxiety tests—open field, light-dark box, EPM) and decreased response to acute stress manifested on FC and SIH tests in Ahi1+/+ mice. In Ahi1+/− mice, CUS did not affect anxiety measures on the open field, light-dark box or EPM. In all three anxiety tests CUS had the potential to induce further effects; thus, the lack of CUS effect in these mice cannot be ascribed to “floor” or “ceiling” effects. Furthermore, CUS did not exert significant effects on sucrose preference, hyperthermic response to acute stress, corticosterone level, or contextual FC in Ahi1+/− mice and did not affect hippocampal neurogenesis as in Ahi1+/+ mice. Taken together, these findings reflect a striking contrast in the effect of CUS in Ahi1+/+ and Ahi1+/− mice and thus represent a significant extension of our previous findings which focused on naive mice not exposed to stress prior to the test situation.

The unexpectedly small SIH effect that we observed may reflect a blunted response to stress following the CUS protocol, possibly reflecting a more resilient phenotype that was promoted by stress in this paradigm. Alternatively, it could reflect a ceiling effect that stems from a higher basal temperature. That CUS induced elevation of corticosterone levels in Ahi1+/+ mice supports the latter. Ahi1+/+ mice exposed to CUS displayed decreased contextual FC but normal cued fear response. This may reflect impaired hippocampal learning^33^ or may be attributed to reduced anxiety^34^. However, the manipulation in both FC and SIH tests is the induction acute stress (i.e., footshock and rectal probe insertion), hence the decreased response of Ahi1+/+ mice on these two tests may reflect blunted response to acute stressors in the context of CUS.

Ahi1+/− mice exposed to CUS displayed higher mobility rate in the FST compared to Ahi1+/− controls (Supplementary Information). In this context, it should be noted that the body mass of Ahi1+/− mice exposed to CUS was significantly higher than all other groups including Ahi1+/− controls. The higher body mass resulted from random assignment, as was evident even before CUS exposure. However, high body mass is known to increase mobility rate^35^, suggesting that the effect on mobility may result from body weight rather than CUS.

Our finding that CUS increased neurogenesis in the dentate gyrus of Ahi1+/+ mice was surprising. Although CUS^36,37^, chronic mild stress^38–40^, and chronic corticosterone treatment^38,41^ are most frequently reported to decrease neurogenesis and number of microglia^36,37^, there are several reports indicating increased neurogenesis following chronic predictable stress^34^ or no effect on neurogenesis following chronic mild stress^42^ and chronic predictable stress^43,44^. Lagace et al.^45^ found that chronic stress may induce anhedonia together with increased neurogenesis. Moreover, it has been demonstrated that decreased neurogenesis is not obligatory for anhedonia in chronic mild stress models^46,47^.

Our chronic stress protocol is a modification of the original protocol of Willner et al.^20^ with emphasis on the unpredictability of the stressors. All the stressors that were employed in this protocol are consistent with a chronic mild stress regimen (e.g.,^38,46,47^). However, the duration of the stress protocol that we employed (9 weeks) was almost twice as long as the protocols reviewed (the longest of them lasted 5–6-weeks). Moreover, the unpredictability of the protocol was greatly enhanced by not only employing stressors in random order as in all unpredictable stress protocols (e.g.,^36–38,46,47^), but by varying the duration of five of the six stressors that were employed. This yielded a unique protocol of mild but highly unpredictable chronic stress. This, combined with the long duration of the protocol, may have contributed to the unique combination of decreased hedonic behavior and increased neurogenesis that we observed. As we have noted, other chronic stress models reported induction of depressive behavior along with increased neurogenesis^45,48^. Moreover, the effect of stress on neurogenesis was demonstrated in stress susceptible but not in stress hyporesponsive animals^45^. Also, stress hyporesponsiveness may gradually develop through the 9-week exposure to CUS protocol. Sucrose preference was measured in the middle of the CUS exposure whereas neurogenesis was measured after termination, 5 weeks later.

The results of the present study suggest that Ahi1 deficiency during neurodevelopment results in attenuated perception and/or integration of environmental stressors but does not affect the response to acute, direct stress as reflected in the normal, stress-induced hyperthermic response that we observed. The minimal response of Ahi1+/− mice to CUS may result from deficient connectivity of the amygdala with other major limbic structures, most prominently the ventral hippocampus and the entorhinal cortex that we described previously^18^. Accordingly, lesion^49^ and muscimol inhibition^50^ of the ventral, but not dorsal, hippocampus reduces anxiety manifested in anxiety tests such as EPM.

Although CUS is usually associated with an increased anxiety response^25,51,52^, several studies have demonstrated decreased anxiety following chronic stress^27–29^. We too have recently demonstrated reduced anxiety following a similar CUS protocol in female mice of an entirely different strain (C57BL/6JRccHsd), suggesting that this effect is neither gender nor strain specific^30^. Moreover, chronic stress has been shown to induce a combination of effects similar to what we have observed—reduced anxiety along with anhedonia^28^. The similarity of the anxiety profile of Ahi1+/+ mice that underwent CUS to the phenotype of Ahi1 deficient mice suggests that chronic stress and Ahi1 deficiency may share a mechanism affecting brain anxiety circuits, such as reduction in amygdalar CRH level. Also, chronic stress may alter the functional connectivity of the brain, gradually altering connectivity of the amygdala with other limbic areas such as ventral hippocampus and lateral entorhinal cortex, similar to our observation of Ahi1+/− mice. Further studies are required to elucidate this hypothesis.

The results of this study should be considered on the background of key limitations. Most notable among these is the fact that contrary to the majority of reported findings, we observed reduced rather than increased anxiety in wild type Ahi1+/+ mice exposed to CUS and also increased rather than reduced hippocampal neurogenesis. In mitigation of this discrepancy we cite other published reports of findings such as these, including or own, and also consider possible factors related to the length and degree of unpredictability of the chronic stress protocol that may have contributed to the ostensibly anomalous findings. Further studies are needed to resolve the discrepancy between our findings and the mainstream of reports and these are in progress in our laboratory. Notwithstanding the discrepant nature of our findings regarding the effects of CUS on anxiety and neurogenesis, our central hypothesis regarding stress hyporesponsiveness in Ahi1+/− mice was supported by the results of our study. To further elucidate the role of Ahi1 in stress responsiveness testing the effect of a CUS protocol on Ahi1 overexpressing mice may provide significant insight as well.

In conclusion, we have shown that CUS administered over 9 weeks induced significant behavioral effects in Ahi1+/+ mice, but had very little effect on Ahi1+/− mice. An effect of CUS on neurogenesis was observed only in Ahi1+/+ mice. As we hypothesized on the basis of our earlier studies^19^, the current data suggest that Ahi1+/− mice may not be stress resilient but rather stress hyporesponsive. This may render them resistant not only to the hazards of stress but also to its potentially beneficial effects. We have suggested that under-expression of Ahi1 during neurodevelopment may lead to deficits in neural connectivity that have a detrimental effect on cognitive-emotional processing^19^. Ostensible stress-resilience may in fact reflect impairment akin to defects in reality testing and/or emotional blunting seen in patients with schizophrenia and possibly autism. A wide spectrum of apparently distinct neuropsychiatric disorders have been suggested to share major genetic components^53^, and impaired cognitive-emotional processing has been suggested as an intermediate phenotype shared by many of these disorders^54^. Thus, the stress-hyporesponsiveness demonstrated by our data offers a potential clue to neural mechanisms that could link Ahi1 with a wide range of neuropsychiatric disorders in humans.

## Electronic supplementary material

Supplementary information
