# Supplementary material for: Effect of chronic unpredictable stress on mice with developmental under-expression of the Ahi1 gene: behavioral manifestations and neurobiological correlates
Source: Transl Psychiatry. 2018 Jul 2;8:124. doi: 10.1038/s41398-018-0171-1 (PMC6028478; doi:10.1038/s41398-018-0171-1)
Supplement: Supplementary file 1 — Supplementary information [file 41398_2018_171_MOESM1_ESM.docx]

***Effect of chronic unpredictable stress on mice with developmental under-expression of the Ahi1 gene: Behavioral manifestations and neurobiological correlates.***

***Wolf et al.***

**SUPPLEMENTARY INFORMATION**

**Supplementary Methods**

**Chronic unpredictable stress (CUS) protocol**


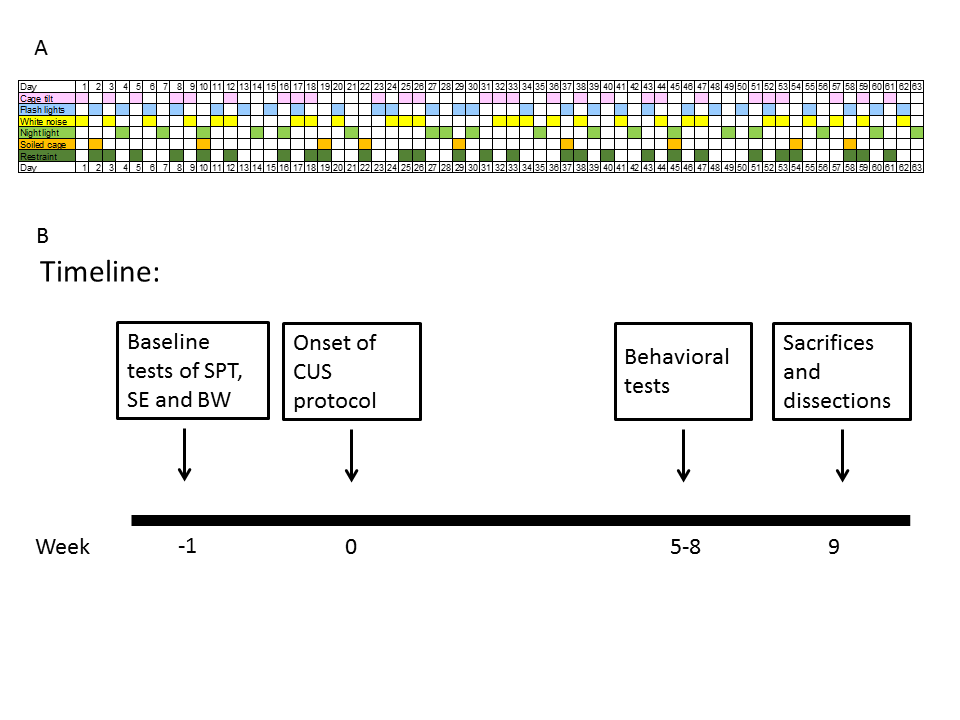


**Fig. S1**: *Top*: Visualization of distribution of stressors of CUS protocol. *Bottom*: Visualization of experimental timeline.

**Further details of behavioral tests**

***Forced swim test (FST):*** Mice were individually placed in a 21 (diameter)*46 (height) cm circular transparent plexiglass tank filled with 15 cm of room-temperature water. During the 6 minutes test time, the time in which mice were actively swimming (mobility) and the time of passive floating (immobility) were measured.

***Social exploration (SE) test***: Mice were individually place in a plastic cage for 15 minutes' habituation, thereafter presented with a male juvenile mouse (~3 weeks old). The amount of time spent by each test animal sniffing the juvenile was recorded. Each animal underwent this test before the beginning of the CUS protocol, after 5 weeks of CUS just before the 3 weeks behavioral tests battery commenced, and after the end of the behavioral tests battery just prior to sacrifice.

***Novel object recognition (NOR) test:*** This test is consisted of two parts- sample object exposure and novel object test. For sample object exposure mice were placed in 25*25 cm arena, containing two identical objects for 10 minutes and then return to their home cages for 1 hour. Thereafter, they were again introduced to the same arena that now contained the sample object and a novel object for 4 minutes test time. The ratio of exploration time of the novel object to the total exploration of both objects time is the discrimination index.

**Fig. S1**: *Top*: Visualization of distribution of stressors of CUS protocol. *Bottom*: Visualization of experimental timeline.

***Table S1: Order of test administration***:

| **Test** | **Battery week** | **Category** |
| --- | --- | --- |
| Sucrose preference (SPT) | Performed twice: before the beginning of CUS protocol and after 4 weeks of CUS. | Depression |
| Social exploration (SE) | Performed 3 times: before the beginning of CUS protocol, after 4 weeks of CUS, and during the 3^rd^ week of the battery (after FC test). | Depression |
| Open field (OFT) | Week 1 | Anxiety |
| Dark-light box (DLB) | Week 1 | Anxiety |
| Novel object recognition (NOR) | Week 1 | Cognitive |
| Elevated plus maze (EPM) | Week 2 | Anxiety |
| Forced swim test (FST) | Week 2 | Depression |
| Fear conditioning (FC) | Week 3 | Cognitive |
| Stress-induced hyperthermia (SIH) | Week 3 | Anxiety |

The table summarize all tests performed in the behavioral battery and the order in which they were performed. Generally, the tests were conducted from the least stressogenic to the most stressogenic, to avoid contamination of the results.

**Corticosterone assay**

Corticosterone levels were measured in serum obtained from mice upon sacrifice. Blood collected from mice was left to clot for 1 hour in room temperature. Next, vials were centrifuged in 4500 rpm and serum was kept in -80 0C until further procedure. Corticosterone level was measured using corticosterone ELISA kit and protocol (R&D systems, Inc., Minneapolis, USA). Briefly, prior to corticosterone assay samples were pretreated and diluted. Next, corticosterone primary antibody solution was added to each well (excluding NSB wells) and incubated for 1 hour at room temperature on horizontal orbital shaker set at 500rpm (Biotest Ltd. Kfar Saba, Israel). Next, well were washed and pretreated and either standard or sample were added to the appropriate wells. Corticosterone conjugate was added to all wells and incubated for 2 hours on the shaker (500rpm). After 2 hours wells were washed, added substrate solution and incubated for 30 min. Next, stop solution was added to each well and optical density was determined immediately in an ELISA reader set to 450 nm with wavelength correction set to 570nm.

Calculation of results were done by ELISA Analysis free online software (<http://www.elisaanalysis.com/>) using 4 parameter logistic curve fit and corticosterone concentrations were multiplied by dilution factor (2).

**Supplementary results**

**Effect of Ahi1 genotype and CUS on body weight**

**Fig. S2**: Body weight gain during the 10 week experimental period. Two-way ANOVA with repeated measures revealed a significant effect of time (F[1,63]=92.574, p=5.52*10^-14^) but not of genotype and no interaction, indicating that mice of all groups gained to a similar extent.

As expected with young adult mice there was a significant increase in body weight during the 9 week experimental period. Because two-way ANOVA revealed a significant genotype by CUS interaction (F[1,63]=6.505, p=0.013) that resulted from higher baseline body weight of CUS Ahi1+/- mice compared to all other groups an ANCOVA was performed for the assessment of body weight change throughout the experimental period, using baseline body weight as a covariate. The contribution of basal bodyweight was statistically significant (F[1,62]=66.332, p=2.223*10^-11^), but no significant contribution of genotype or CUS was demonstrated.

**Effect of genotype and CUS in the forced swim test**

Ahi1+/- mice displayed higher mobility in the forced-swim test compared to Ahi1+/+ mice, manifested by a significant main effect of genotype (F[1,69]=6.33, p=0.014) on two-way ANOVA. Interestingly, CUS further increased mobility of Ahi1+/+ and Ahi1+/- alike, reflected in a significant main effect of CUS (F[1,69]=9.568, p=0.003). No significant genotype by CUS interaction was observed. Post hoc comparisons of simple main effects indicated higher mobility of Ahi1+/- CUS compared to Ahi1+/+ CUS mice (p<0.05) and Ahi1+/- controls (p<0.005).

**Effect of genotype and CUS on social exploration**

**Fig. S3**: Effect of CUS on social exploration. Two-way ANOVA with repeated measures revealed a between-subject effect of genotype (F[1,63]=18.017, p=0.00007), indicating that Ahi1+/- mice displayed longer social exploration duration. No significant within subject effects of CUS or genotype were observed, indicating that CUS exposure did not reduce social exploration.

Ahi1+/- mice displayed a longer duration of social exploration (Fig. S-G-2), reflected by a significant between-subject effect of genotype (F[1,63]=18.017, p=0.00007) on two-way ANOVA with repeated measures. No significant effect of CUS or CUS by genotype interaction were observed.

**Effect of Ahi1 genotype and CUS on novel object recognition**

Ahi1+/+ and Ahi1+/- mice displayed a similar discrimination ratio in the novel object recognition test. Accordingly, no significant effect of CS, genotype, or CS by genotype interaction was demonstrated in a two-way ANOVA.

**
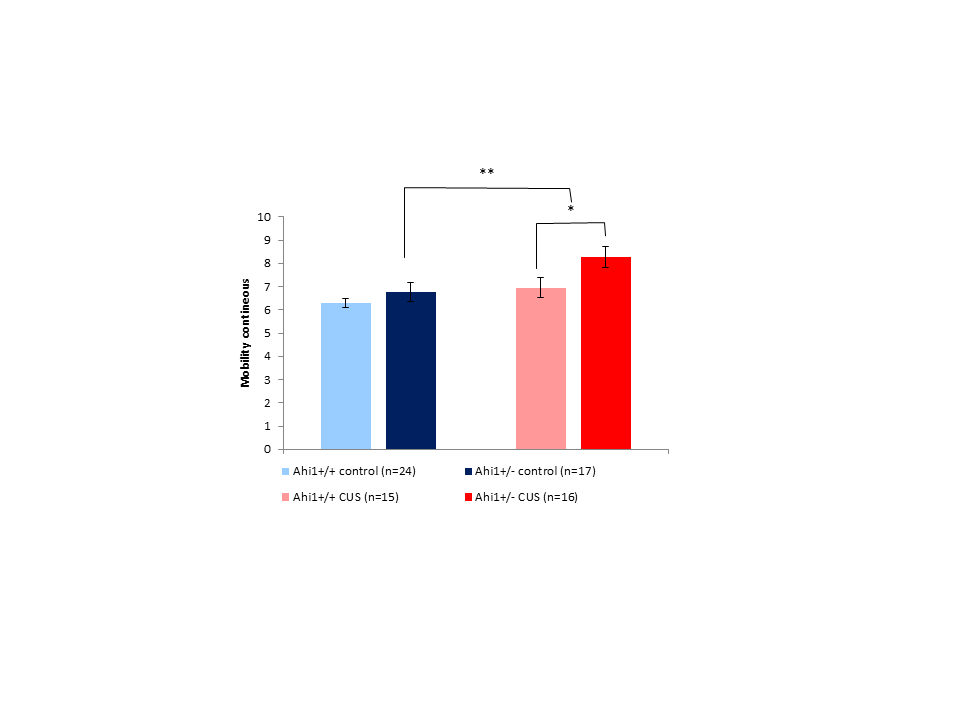
**

**Fig. S4**: Effect of CUS on mobility in the forced swim test. Two-way ANOVA revealed a significant main effect of genotype (F[1,69]=6.33, p=0.014), reflecting higher mobility of Ahi1+/- mice. CUS further increased the mobility of both Ahi1+/+ and Ahi1+/- mice, identified in a significant main effect of CUS (F[1,69]=9.56, p=0.003). Post hoc comparisons of simple main effects indicated higher mobility of Ahi1+/- CUS compared to Ahi1+/+ CUS mice (p<0.05) and to Ahi1+/- controls (p<0.005). *p<0.05, **p<0.005
